# Social Service Utilization, Sense of Community, Family Functioning and the Mental Health of New Immigrant Women in Hong Kong

**DOI:** 10.3390/ijerph10051735

**Published:** 2013-04-29

**Authors:** Qiaobing Wu, Julian Chun-Chung Chow

**Affiliations:** 1 Department of Social Work, The Chinese University of Hong Kong, Shatin, NT, Hong Kong; 2 School of Social Welfare, University of California, Berkeley, CA 94720, USA; E-Mail: jchow99@berkeley.edu

**Keywords:** depression, family functioning, new immigrant women, sense of community, social service utilization

## Abstract

Drawing upon a sample of 296 new immigrant women in Hong Kong, this study investigated how social service utilization, family functioning, and sense of community influenced the depressive symptoms of new immigrant women. Results of the structural equation modeling suggested that family functioning and sense of community were both significantly and negatively associated with the depression of new immigrant women. Utilization of community services also influenced the depression of immigrant women indirectly through the mediating effect of sense of community. Implications of the research findings for mental health intervention were discussed.

## 1. Introduction

New immigrants from the mainland China constitute a considerable proportion of the entire population of Hong Kong. Under Article 22 of the Basic Law of HKSAR, residents of the mainland may come to Hong Kong for resettlement through the One-Way Permit (OWP) scheme. Mainly for the purpose of family reunion, the OWP scheme allows non-resident spouses and children to join their families in Hong Kong, with a quota of 150 persons per day. From 1997 to 2001, new immigrants coming from mainland China through the OWP scheme had contributed to 93% of Hong Kong’s population growth [1]. Given that sixty percent of the daily quota in the OWP scheme is designated for family unification, most of the new immigrants are children or wives of Hong Kong residents [2]. According to the statistics of the Home Affairs Department and the Immigration Department [3], a total of 210,065 new immigrants arrived in Hong Kong through the OWP scheme; of these, 75.9% were women aged 15 years or above. 

A number of studies have demonstrated the difficulties of new immigrant women in adapting to the culture and life in Hong Kong. They are faced with financial difficulties, language barriers, cultural differences, employment difficulties, problems in family relationships, lack of social support, and discrimination from the mainstream society, all of which affect their adjustment and mental health [4,5,6,7,8,9,10,11,12]. According to statistics of the Home Affairs Department and Immigration Department [13], the median household income of new immigrant families is HK$7,900, less than even half of the median of all households in Hong Kong. Over fifty percent of new immigrants live in public housing. The greatest difficulties they encounter in adapting to life in Hong Kong include employment, living environment, language and family finances, *etc.* With the hope of obtaining more opportunities and improving living conditions by migration, the discrepancies between their expectation of the life in Hong Kong and the reality create additional threats to their psychological wellbeing [14]. However, little research has been done to investigate which factors in their ecological contexts, such as in the family and community, influence the mental health status of new immigrant women. 

Considerable research in the Western literature has documented the significant association between mental health and family functioning [15,16,17]. This is evident in many studies of immigrant families as well. For example, Sarmiento and Cardemil’s study of low-income Latino families revealed a significant association between family functioning and depression, and this association is especially strong in women [18]. In a study of Central American immigrants, Hovey finds that family functioning, together with hopefulness towards the future and socioeconomic status, can serve a protective function against depression of this population [19]. Another study on Mexican migrant farm workers in the Midwest also reported significant association between family dysfunction and higher levels of depression [20]. 

On the other hand, at the neighborhood level, sense of community constitutes another important factor that may shape the living and perceptual experiences of immigrants. As articulated by McMillan and Chavis [21], sense of community entails four dimensions, including needs fulfillment (a perception that members’ needs will be met by the community), group membership (a feeling of belonging or a sense of interpersonal relatedness), influence (a sense that one matters, or can make a difference, in a community and that the community matters to its members), and emotional connection (a feeling of attachment or bonding rooted in members’ shared history, place or experience). Studies have found the sense of community of immigrants to be associated with a series of individual and contextual factors, as well as their health and mental health status [22,23]. For example, Davidson and Cotter’s [24] study with three random samples in South Carolina and Alabama suggests that sense of community is significantly associated with the subjective well-being of all three samples, including their happiness, worrying and personal coping. Prezza and colleagues’ [25] study with 630 residents in Rome, Italy, find sense of community to be associated with their loneliness and life satisfaction regardless of their area of residence. Based on a representative sample of 941 Hong Kong Chinese through a randomized household survey, Mak, Cheung, and Law [26] also find that sense of community is negatively associated with daily hassles and positively associated with social support and quality of life. However, to the authors’ knowledge, no empirical studies have examined the mental health of new immigrant women in Hong Kong in relation to these family and community factors. The present study will thus investigate how family functioning of new immigrant women and their sense of community in the host city would influence the level of their depression. 

Another factor that might affect the mental health of new immigrant women is their utilization of social services. To facilitate the adaptation of new immigration to the new social environment in Hong Kong, various kinds of social services are currently provided by both government and non-governmental organizations, including employment services, medical services, education support, legal aids, family assistances, and community integration [27]. For example, the Labor Department and Employees Retraining Board provide all kinds of employment-related information and job training programs for immigrants. The Integrated Family Services Centers and Integrated Youth Services Centers also provide a variety of counseling services for immigrant families and their young kids. However, little is known about the effects of social service utilization on the mental health of new immigrant women in Hong Kong. Even less known is the mechanism by which social service utilization function on immigrant women’s mental health. 

**Figure 1:**
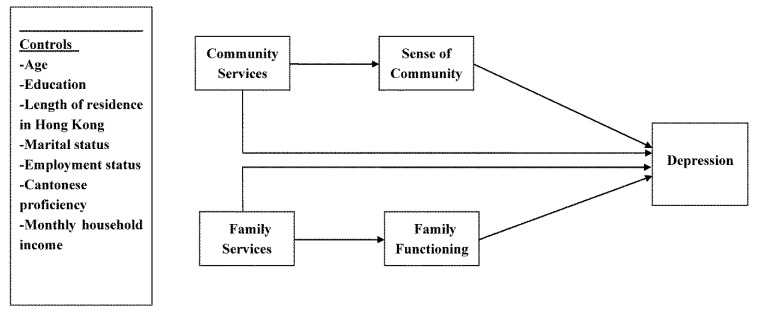
Hypothesized model of social service utilization, family functioning, sense of community and depression.

To fill the gaps in the existing literature, this study aims to investigate whether and how utilization of social services by new immigrant women in Hong Kong, particularly family services and community services, influences their mental health status, especially through the mediating effects of family functioning and sense of community. It is hypothesized that: (1) family functioning of new immigrant women will influence their mental health status; (2) family functioning of new immigrant women will also mediate the effect of utilizing family services on their mental health status; (3) immigrant women’s sense of community will influence their mental health status; and (4) immigrant women’s sense of community will also mediate the effect of utilizing community services on their mental health status. The conceptual framework of the study is presented in Figure 1. 

## 2. Methods

### 2.1. Participants and Procedure

Given the limitation of resources, participants of the study were recruited through convenient sampling. In December 2011, researchers set a booth in the residential community of each of the three districts in Hong Kong, Tin Shui Wai, Tsuen Wan and Sham Shui Po, where new immigrants are mainly concentrated. Women who passed by the booths were invited to participate in the study if they met the eligibility criteria and were willing to participate. The eligibility criteria included: (1) age 18 years or above; (2) had lived in Hong Kong for less than seven years with a One Way Permit, or had lived in Hong Kong for at least three years, but still waiting for the One Way Permit. Invited participants were first provided with the consent form so that they knew about the basic information of the research and knew that their participation was completely voluntary. Recruited participants who signed the consent forms were then asked to complete a paper and pencil questionnaire with the assistance of our research staff. Considering that these immigrant women came from mainland China and had limited knowledge of Cantonese and traditional Chinese, simplified Chinese was used in the questionnaire. They were also encouraged to ask our research staff for clarification if they did not understand certain questions. It took about thirty minutes to complete each survey. Participants who successfully completed the survey were each provided with a supermarket voucher as compensation for their time and efforts. A total of 296 new immigrant women were recruited for study through this approach. 

### 2.2. Measures

*Depression* was measured by the Center for Epidemiological Studies-Depression Scale (CES-D) [28], which assessed contemporary depressive symptoms. Respondents were asked to describe how many days during the past week they experienced the symptom stated in each item, such as “did you have trouble shaking off sad feelings”, “did you feel sad”, and “did you feel that your life had been a failure”. Each item provided a 5-point response scale that included “0 days”, “1 day”, “2–3 days”, “4–5 days”, and “6–7 days” with higher scores indicating higher depression. The sum score of all 20 items was computed to represent the degree of depression in data analysis. The CES-D has demonstrated good psychometric properties across populations and across cultures [29]. The Chinese version of CES-D has been used in studies with Chinese adults and adolescents and demonstrated good validity and reliability [30]. The Cronbach’s *α* of the CES-D in this sample was 0.91. 

*Family Functioning* was assessed by the General Functioning subscale of the McMaster Family Assessment Device (FAD) [31]. The General Functioning subscale had 12 items, six of which described the family in a positive way and six negative. Each item was responded to on a scale from 1 (=totally disagree) to 4 (=totally agree), allowing participants to rate on the degree to which they agree on the statements when considering the real situation of their families. Sample items include “we can express feelings to each other”, “we don’t get along well together”, *etc.* The Chinese version of the General Functioning subscale of the FAD had been used in studies with the Chinese population and demonstrated good validity and reliability [32]. The final family functioning score used in analysis was computed as the sum score of all items (negative statement items reverse-coded first), with higher scores indicating better family functioning. The Cronbach’s *α* of the scale in this sample was 0.809. 

*Sense of Community* was measured by the 8-item Brief Sense of Community Scale (BSCS) [33]. It was designed to assess four dimensions of the sense of community, including needs fulfillment, group membership, influence, and emotional connection [21]. Each item was responded to on a 5-point scale ranging from 1 (totally disagree) to 5 (totally agree). Sample items included “This neighborhood helps me fulfill my needs”, “I belong in this neighborhood”, “I have a good bond with others in this neighborhood”, *etc.* The sum score of all eight items was computed to represent the degree of sense of community in data analysis, with higher scores indicating stronger sense of community. The Cronbach’s *α* of the BSCS in this sample was 0.776.

*Social Service Utilization* was assessed by the frequencies of utilizing family and community services, where family services included family life education, parent-child activities, self-help groups, *etc.*, while community services included programs promoting the adaptation of new immigrants in the community, seminars, travels, and carnivals, *etc.* Participants were asked to rate on a scale from 0 to 6 to describe how often they utilized family and community services respectively, with 0 representing never, 1 representing once a week, 2 representing once every other week, 3 representing once a month, 4 representing once every three months, 5 representing once every 6 months, and 6 representing once a year of less. 

The socio-demographic variables included in the test of the hypothesized model included age, education, marital status, employment status, income, Cantonese proficiency, and length of stay in Hong Kong. Marital status (1 = married; 0 = others) and employment status (1 = working full-time or part-time currently; 0 = currently not working) were both dummy coded. Education was measured on seven categories, ranging from “did not go to school or did not graduate from primary school” (1) to “university degree or higher” (7). Monthly household income was also measured on seven categories, ranging from “HK$4,999 or below” (1) to “HK$30,000 or above” (7). Proficiency of Cantonese was measured on a 5-point scale ranging from “do not understand” (1) to “very good” (5). Length of stay was measured by the number of months they had lived in Hong Kong by the time they completed the survey. 

### 2.3. Data Analysis

Structural equation modelling (SEM) was conducted using MPlus 5.0 [34] to test the hypothesized model. Given the hypothesized direct and mediating effects of service utilization, family functioning and sense of community, SEM is a well-suited statistical technique for this study because it permits the simultaneous estimation of direct and indirect paths and estimates each path after accounting for the effects of all other paths. Multiple indices were used to assess the goodness of fit of the model, including: (1) the likelihood ratio test statistic (χ^2^), where an associated probability value showing non-significant χ^2^ represents a closer fit of the hypothesized model to the perfect fit; (2) the Comparative Fit Index (CFI), where values above 0.90 denote a good model fit; and 3) the Root Mean Square Error of Approximation (RMSEA), where values less than 0.05 indicate a “close fit” [35]. 

## 3. Results

### 3.1. Descriptive Analysis of Sample Characteristics

A total of 296 new immigrant women participated in the present study. The age of the participants range from 22 to 68 years, with a mean of 37.8 years (SD = 8.3). Over eighty percent (83.1%) of these immigrant women were not employed, and over half (54.3%) of the participants reported less than HK$10,000 household income per month, where the median household income of Hong Kong in 2011 was HK$ 20,200. Full details of the sample characteristics are presented in Table 1. 

**Table 1:** Descriptive statistics of sample characteristics.

	Frequency (N)		Percent (%)
**Age**	Mean = 37.83 (SD = 8.26) (years)		
**Length of Residence in Hong Kong**	Mean = 43.05 (SD = 28.07) (months)		
**Marital Status**			
Married	271	91.6	
Others	25	8.4	
**Employment Status**			
Currently working	44	14.9	
Currently not working	251	85.1	
**Education **			
Didn’t attend or finish elementary school	10	3.4	
Elementary school	51	17.2	
Junior high school	156	52.7	
Senior high school	58	19.6	
Professional/vocational school	13	4.4	
Diploma	8	2.7	
**Cantonese Proficiency**			
Do not understand	67	22.7	
Poor	75	25.4	
Fair	125	42.4	
Good	27	9.2	
Excellent	1	0.3	
**Monthly Household Income**			
HK$4,999 or below	50	17.1	
HK$5,000–9,999	109	37.2	
HK$10,000–14,999	102	34.8	
HK$15,000–19,999	21	7.2	
HK$20,000–24,999	11	3.8	

### 3.2. Test of Structural Model

Test of the hypothesized structural model yielded a perfect fit to the data. It generated a non-significant Chi Square value (χ^2^ = 1.895, df = 2, p = 0.388), a CFI of 1.000 and a RMSEA of 0.000. A total of 45.1% of the variance in depression was explained by this model. 

**Figure 2:**
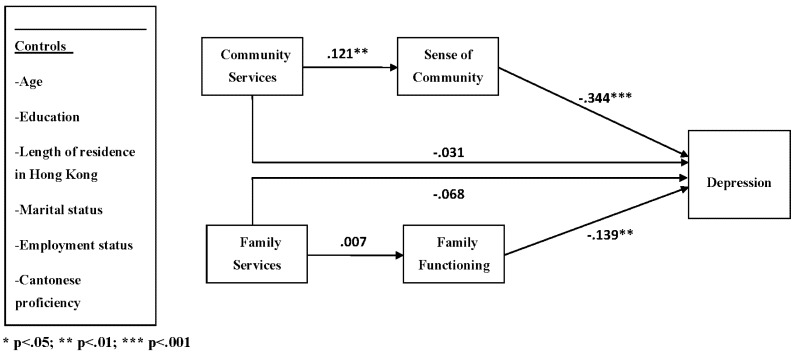
Standardized solutions for thestructural model of social service utilization, family functioning, sense of community and depression.

As hypothesized, family functioning and sense of community both exhibited significant negative effects on the depression of new immigrant women. Depression appeared to be at a lower level when there were better family functioning (β = −0.139, *p* < 0.01) and stronger sense of community (β = −0.334, *p* < 0.001). The two social service utilization variables, use of family services (β = −0.068, *p* > 0.05) and use of community services (β = −0.031, *p* > 0.05) did not show significant direct effects on the depression of immigrant women. However, utilization of community services did influence women’s depression though the mediating effect of the sense of community. New immigrant women who used community services more frequently tended to present stronger sense of community (β = 0.121, *p* < 0.01), which, in turn, led to lower level of depression. Nevertheless, this indirect effect did not appear for family services, where the frequency of utilizing family services was not significantly associated with the degree of family functioning (β = 0.007, *p* > 0.05). The standardised solution for the test of the structural model is presented in Figure 2.

Of the socio-demographic variables, education attainment and proficiency of Cantonese showed significant direct effects on immigrant women’s depression. The higher the women’s education, the higher the level of their depression (β = 0.408, *p* < 0.001); while the better the women’s Cantonese proficiency, the lower the level of their depression (β = −0.102, *p* < 0.05). Education also influenced women’s depression through the mediating effects of family functioning and sense of community. Higher educational attainment was associated with worse family functioning (β = −0.266, *p* < 0.001) and weaker sense of community (β = −0.206, *p* < 0.01), which, in turn, predicted higher degree of depression. In addition, sense of community also mediated the effect of Cantonese proficiency on immigrant women’s depression. Better Cantonese frequency was associated with stronger sense of community (β = 0.124, *p* < 0.05), thus leading to lower level of depression. The standardized direct, indirect and total effects of major predictor variables on the depression of immigrant women were presented in Table 2. 

**Table 2:** Standardized direct, indirect, and total effects of major predictor variables on immigrant women’s depression.

Major Predictor Variables	Depression		
	Direct	Indirect	Total
Family functioning	−0.139 **	-	−0.139
Sense of community	−0.344 ***	-	−0.344
Utilization of family services	−0.068	−0.001	−0.069
Utilization of community services	−0.031	−0.107 *	−0.138
Age	0.011	0.016	0.027
Length of residence in Hong Kong	0.027	0.029	0.056
Marital status	−0.014	0.027	0.013
Employment status	−0.002	0.026	0.024
Education	0.408 ***	0.108 ***	0.516
Cantonese proficiency	−0.102 *	−0.042 *	−0.144
Monthly household income	0.033	0.024	0.057

*p< 0.05; **p< 0.01; ***p< 0.001.

## 4. Discussion and Conclusions

This study investigates the depression of new immigrant women in Hong Kong in relation to their social service utilization, family functioning and psychological sense of community. Particularly, it investigates how the immigrant women’s utilization of social services might influence their depression through the mediating effects of family functioning and sense of community. The study results reveal the mechanism by which these aforementioned contextual factors operate on the depressive symptoms of immigrant women. Major findings of the research are summarized below with further elaboration and interpretation of each. 

First, consistent with the existing literature, family functioning and sense of community are both associated with less depressive symptoms of new immigrant women in Hong Kong. This is fairly understandable considering the special situation of new immigrant families. Given the fact that many immigrant women come to Hong Kong for family reunion purposes, the average five year waiting period for them to receive the One Way Permit and get a legal residence in Hong Kong forces them to live apart from their spouses and children for a long time. This leads to the lack of communications among family members and the lack of experiences of tackling difficulties together, which increases the risk of developing family conflicts as they start a new lifestyle in a new social environment [29]. Therefore, family is the first domain where immigrant women must make considerable adjustments right after they resettle in Hong Kong, and family functioning would thus become a most influential factor for their mental health. Immigrant women whose family dynamics function in healthier manners are naturally better off in their mental health, as reflected by less depressive symptoms in this study. 

Besides, given that most immigrant women will not be able to find a job immediately, the neighborhoods where they live become an important environment where they spend most of their time in the day. How they perceive their neighborhoods would thus exert significant effects on their mental health as well. The extent to which they feel their needs met by the neighborhood, feel connected to other people in the same neighborhood, and feel belonging to the neighborhood is closely associated with their psychological well-being. Stronger sense of community will help protect immigrant women against developing depressive symptoms. This may derive from the feel of security and support when the neighborhood provides them with a sense of belonging and bonding with others. 

Moreover, sense of community also mediates the effect of utilization of community services according to the research findings, which is of primary interest of the present study. As the study results suggest, more frequent utilization of community services is associated with stronger sense of community, which, in turn, leads to lower level of depression. Currently, community services provided to new immigrants in Hong Kong mainly include seminars on related issues of immigrants and their families, small trips for immigrants to know more about Hong Kong, carnivals in special occasions or holidays, *etc.* Participation in these community programs provides an efficient channel for new immigrant women to become familiar with the neighborhood and provides a platform for them to interact with other people in the neighborhood. As they resettle in the new community, these initial interactions will largely help immigrant women rebuild their social networks destroyed by the migration process. The gradual establishment of new social ties will conceivably strengthen their feeling of connectedness with other people in the neighborhood and fortify their sense of belonging to the neighborhood, which contribute to a stronger sense of community. This explains why sense of community plays a mediating role between community services and the immigrant women’s mental health. However, utilization of family services does not show similar indirect effects through family functioning. This is probably due to the fact that the utilization rate of family services is generally low among new immigrants. As the study results show, over seventy percent (71.3%) of participants never used family services. The low variation of the family service utilization variable may partially explain its non-significant effect on family functioning. 

One interesting finding of the study is the negative effect of immigrant women’s education. Higher level of educational attainment is associated with more depressive symptoms directly as well as with worse family functioning and weaker sense of community, thus predicting higher depression indirectly. This seems against the typical view that human capital, achieved mainly through education, can serve as a protective factor of one’s mental well-being. However, considering the special situation of immigrant women, it is possibly attributed to the discrepancy between their expectations of the life in Hong Kong after family unification and the reality. Women with higher education tend to hold higher expectation of their career development, social status and living conditions as they resettle in Hong Kong. Whereas the reality that they have to live in densely populated immigrant communities with limited employment opportunities, and even face discrimination from the mainstream society, drives them into deep frustration. This frustration caused by the unmatched expectation and reality can be a trigger of depression itself, as well as a barrier to adaptation to the new family and community environment, which leads to family dysfunction and low identification with the community. Therefore, higher education shows unexpected negative effects on the mental health of immigrant women through both direct and indirect pathways. 

To conclude, the present study suggests that, family functioning and sense of community are both influential for the mental health of new immigrant women in Hong Kong. Social service utilization, particularly the use of community services, is important for fostering stronger sense of community, thus alleviating the depressive symptoms of new immigrant women as well. However, these findings should be interpreted with caution because of several limitations of the study. First, due to resource limitation, participants of the study were recruited through convenient sampling. The generalizability of the research findings were thus constrained by this non-probability sampling strategy. Second, with a cross-sectional design, it is hard for the present study to ensure the direction of causal relationships among the major variables tested in the model. For example, it is possible that immigrant women with stronger sense of community tend to use community services more frequently, instead of vice versa. Therefore longitudinal data is needed in future research to further examine the relationship patterns among social service utilization, sense of community, family functioning and mental health of immigrant women. Third, in addition to the variables tested in the model, there are still many other factors that may influence the mental health of immigrant women (e.g., living status, family structure) but are not controlled in the study. Future research is expected to continue exploring potential factors that may have an impact on the mental health of this population.

Despite the above limitations, findings of the research do have several implications for mental health intervention. First, given the significant effects of family functioning, health interventions could focus on family education programs that equip immigrant women and their family with useful skills to facilitate better family communications and deal with family conflicts in more constructive ways. Lowering the chance of family dysfunction will effectively prevent the development of depression among immigrant women. Second, considering the importance of sense of community for the mental health of immigrant women and the indirect effect of utilizing community services through sense of community, interventions should also place considerable efforts on fortifying the bonds among neighbors in immigrant communities and strengthening their sense of belonging to the neighborhood. As the study results suggest, developing more community service programs and encouraging the utilization of such services might be an effective approach to promoting the mental health status of immigrant women. Third, given the significant effect of Cantonese proficiency on the depression of immigrant women either directly or through the mediating effect of sense of community, providing language programs for new immigrant women for them to adapt to the new social environment more easily could be a potentially effective mental health intervention as well.
